# Crosstalk between bone and the immune system

**DOI:** 10.1007/s00774-024-01539-x

**Published:** 2024-07-26

**Authors:** Kazuo Okamoto

**Affiliations:** 1 Department of Osteoimmunology, Graduate School of Medicine and Faculty of Medicine, The University of Tokyo, Tokyo, Japan; 2 Division of Immune Environment Dynamics, Cancer Research Institute, Kanazawa University, Kakuma-Machi, Kanazawa, 920-1192 Japan

**Keywords:** Bone, Immune system, Osteoimmunology, Osteoclast, RANKL, Bone marrow microenvironment

## Abstract

Bone functions not only as a critical element of the musculoskeletal system but also serves as the primary lymphoid organ harboring hematopoietic stem cells (HSCs) and immune progenitor cells. The interdisciplinary field of osteoimmunology has illuminated the dynamic interactions between the skeletal and immune systems, vital for the maintenance of skeletal tissue homeostasis and the pathogenesis of immune and skeletal diseases. Aberrant immune activation stimulates bone cells such as osteoclasts and osteoblasts, disturbing the bone remodeling and leading to skeletal disorders as seen in autoimmune diseases like rheumatoid arthritis. On the other hand, intricate multicellular network within the bone marrow creates a specialized microenvironment essential for the maintenance and differentiation of HSCs and the progeny. Dysregulation of immune–bone crosstalk in the bone marrow environment can trigger tumorigenesis and exacerbated inflammation. A comprehensive deciphering of the complex “immune–bone crosstalk” leads to a deeper understanding of the pathogenesis of immune diseases as well as skeletal diseases, and might provide insight into potential therapeutic approaches.

## Introduction

Bone is the key component of the locomotor system that supports and moves the body. The immune system is the host defense system that eliminates foreign substances such as microorganisms. Bone and the immune system may seem completely unrelated, but in fact, they interact and cooperate with each other in various physiological and pathological settings. Bone-resorbing cells osteoclasts belong to the monocyte/macrophage lineage, and receptor activator of NF-κB ligand (RANKL) is essential for immune organ development as well as osteoclast differentiation. Immune cells and bone cells share many regulatory factors such as cytokines, chemokines and receptors. Thus, the effects of abnormal immune activation such as autoimmunity can spill over into the skeletal system. Osteoimmunology was created and has developed as a new interdisciplinary field focusing on the crosstalk between the skeletal and immune systems [1]. The term “osteoimmunology” was first used in an editorial article in the *Nature* in 2000 in response to a paper on osteoclast regulation by T cells [2]. Since then, a variety of bone-immune interactions have been widely elucidated, not limited to the interaction between osteoclasts and T cells. Immune cells circulate in the body through blood and lymph vessels to peripheral tissues. Therefore, given the fact that immune cells attack a variety of tissues as seen in inflammatory diseases, it is evident that bone is not the only organ with which the immune system interacts. However, bone marrow is the primary lymphoid organ that provides the environment for differentiation and maintenance of hematopoietic stem cells (HSCs) and immune progenitor cells [3]. It should be noted that not only do the immune cells affect bone, but also bone cells regulate the immune system. In this review, I provide a comprehensive overview of the mutual relationship between the skeletal and immune systems in health and diseases by introducing the key background as well as the latest research progress.

## RANKL, a representative factor linking bone and immunity

RANKL was reported in 1998 as a cytokine essential for osteoclast differentiation [4, 5], but it had already been identified in immunology the year before as a novel TNF family cytokine expressed on T cells [6, 7]. RANKL is a multifunctional cytokine that plays essential roles in not only bone but also immune regulation. Thus, RANKL is one of the most important osteoimmune molecules explicitly linking the skeletal and immune systems.

### The cellular source of RANKL in bone remodeling

**Fig. 1:**
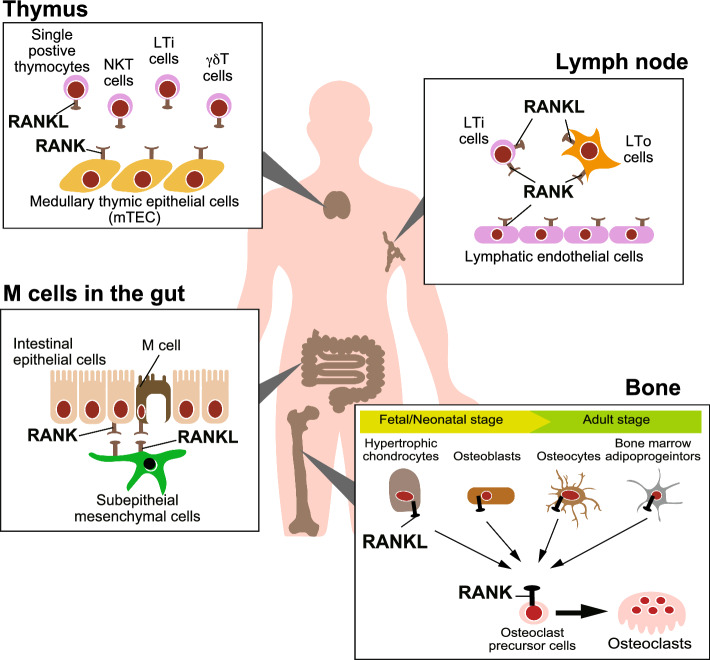
Critical role of RANKL in the immune organ development. RANKL plays essential roles in the development of the immune organs—bone marrow, thymus, lymph nodes and gut-associated lymphoid tissues. In each tissue formation, various cell types act as sources of RANKL expression. Regarding skeletal development and bone remodeling, the main cellular source of RANKL for osteoclast differentiation varies by life stages; hypertrophic chondrocytes and osteoblasts in fetal and neonatal stages, and osteocytes and bone marrow adipoprogenitors cells in adult stages

Based on analyses of cell type-specific RANKL-deficient mice, it has been shown that hypertrophic chondrocytes and osteoblasts are the major sources of RANKL in endochondral ossification during embryonic and growth stages, whereas osteocytes act as the main source of RANKL in adults [1, 8, 9]. RANKL expression in osteocytes is caused by mechanical loading, cellular senescence and cell death [10, 11]. In addition, bone marrow adipose progenitor cells labeled with Adiponectin-Cre have been shown as a source of RANKL for controlling trabecular bone mass in adults [12, 13] (Fig. 1). On the other hand, osteoprotegerin (OPG), a decoy receptor for RANKL, is mainly produced by newly formed osteocytes near the surface of cortical bone [14]. Long-term treatment with anti-RANKL antibody denosumab decreased new osteocytes and OPG production, contributing to rebound resorption after denosumab discontinuation.

### Roles of RANKL in the immune system

RANKL is essential for the differentiation of medullary thymic epithelial cells (mTECs). The main source of RANKL-producing cells for mTEC differentiation are single positive thymocytes after birth [15]. During ontogeny, lymphoid tissue inducer (LTi) cells, γδ T cells and natural killer T (NKT) cells express RANKL for mTEC development [1]. Furthermore, RANKL-induced mTEC differentiation was involved in the expansion of natural regulatory T (Treg) cells in pregnancy. Thymic deletion of the *Tnfrsf11a* gene (encodes RANK, the receptor for RANKL) caused the reduced accumulation of natural regulatory T (Treg) cells in the placenta and visceral adipose tissue, resulting in miscarriages and gestational diabetes [16]. RANKL- and RANK-deficient mice also showed a systemic loss of lymph node formation [17]. Activation of RANK^+^ lymphatic endothelial cells by LTi cells via RANKL in the lymphoid anlagen, followed by activation of mesenchymal lymphoid tissue organizer (LTo) cells, is necessary for lymph node organogenesis [18]. In addition, RANKL signal is required for microfold cell (M cell) differentiation in the gut. Subepithelial mesenchymal cells of the gut-associated lymphoid tissues induce M cell differentiation by supplying RANKL to RANK-positive intestinal epithelial cells [19]. Given that osteoclasts are required for the formation of the bone marrow cavity, RANKL could be considered a cytokine for the development of immune organs (Fig. 1).

T cell-derived RANKL does not contribute to bone metabolism but triggered chronic inflammation of the central nervous system by interacting with RANK-expressing astrocytes in multiple sclerosis [20]. In the tumor microenvironment of breast cancer, RANKL expressed on Treg cells stimulated tumor cells to promote the metastatic activity [21]. Moreover, RANKL on epidermal cells induces Treg cell proliferation via Langerhans cells, suppressing inflammatory skin diseases. The RANKL/RANK system exerts immunological functions not only in the immune organ development but also in various pathological conditions including inflammatory diseases and cancer.

### Local regulation of the RANKL/RANK system

RANKL is synthesized as a membrane-bound molecule, which is cleaved into the soluble form by proteases. Although both forms function as agonistic ligands for RANK, soluble RANKL-deficient mice did not show any discernible osteopetrotic phenotype [22]. In addition, soluble RANKL did not contribute to pathological osteoclast activation in ovariectomy-induced osteoporosis and periodontitis [22, 23], suggesting that membrane-bound RANKL mainly functions in osteoclast differentiation in vivo. Furthermore, unlike RANKL-null mice, soluble RANKL-deficient mice had normal development of the immune organs, including mTEC differentiation, lymph node organogenesis and M cell differentiation [22]. Thus, soluble RANKL is dispensable for physiological regulation of bone and immune systems. OPG also circulates in the blood as a soluble protein. Analyses of osteoblast-specific, thymic epithelial cell-specific and intestinal epithelial cell-specific OPG-deficient mice have revealed that bone remodeling, mTEC differentiation and M cell differentiation is controlled by OPG locally produced in bone, thymus and intestine, respectively, but not by circulating OPG [24]. The findings further revealed the importance of local regulation of the RANKL/RANK system.

## Osteoclasts, key hematopoietic lineage cells involved in the skeletal system

Osteoclast is one of the crucial hematopoietic lineage cells in bone, and studies on mechanism of osteoclast differentiation have highlighted the similarities between bone and immune cells. Osteoclasts are large multinucleated cells formed by the fusion of myeloid lineage precursor cells. HSCs support hematopoiesis throughout life after birth and give rise to a wide variety of immune cells. However, recent findings indicate that tissue-resident macrophages such as Kupffer cells in the liver and microglia in the brain originate from the primitive erythro-myeloid progenitors (EMPs) in the yolk sac at the early embryonic stage [25]. Regarding osteoclasts, yolk sac-derived EMPs give rise to osteoclasts during embryonic and fetal development, whereas osteoclasts arise from HSC-derived monocyte/macrophage precursor cells in postnatal life [26, 27]. Osteoclast differentiation is induced by stimulation of RANKL, which is produced by mesenchymal cells such as osteoblastic lineage cells [1, 17]. Downstream of its receptor RANK, NF-κB and mitogen-activated protein kinases (MAPKs) are activated via TNF receptor-associated factor (TRAF) 6 and induce the expression of nuclear factor of activated T cells (NFATc1) [1, 28]. NFATc1 was originally identified as a transcription factor for T cell activation, but in osteoclasts, it functions as the master regulator for osteoclastogenesis. c-Fos is also induced by RANKL stimulation via CREB and NF-κB, and crucially involved in NFATc1 induction as the AP-1 dimer formed with Jun proteins [1, 28–31]. Notably, c-Fos is known to play a critical role in osteosarcoma development as well as in the regulation of gene expression downstream of immune-related signals such as antigen receptors and cytokine receptors. NFATc1 directly induces the expression of genes related to cell morphological changes, fusion and bone resorption activity of osteoclasts, cooperating with other transcriptional factors such as AP-1, MITF and PU.1 [1]. In addition, signals from immunoglobulin-like receptors associated with DAP12 and FcRγ act as co-stimulators for RANK signaling, leading to activation of Tec and Btk, known as important tyrosine kinases downstream of antigen receptors in lymphocytes. Tec and Btk in turn trigger calcium signaling essential for induction and activation of NFATc1 [1]. Thus, molecules with well-known functions in immune cells were unexpectedly found to play critical roles in osteoclast differentiation. Studies on RANKL signaling in osteoclasts have revealed striking similarities between bone and immune cells.

## Regulation of bone by the immune system

### Immune-bone crosstalks in rheumatoid arthritis

**Fig. 2:**
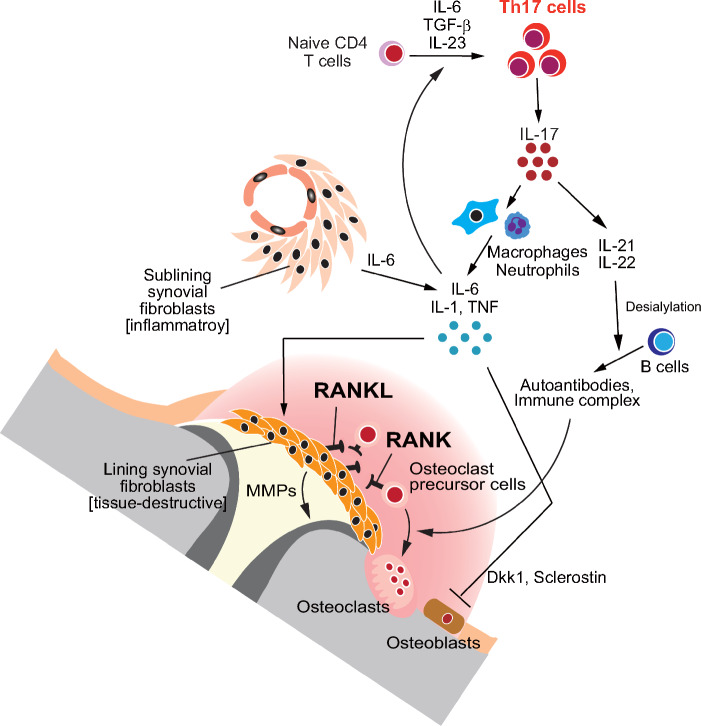
Mechanism of bone destruction in RA. The intricate immune–bone interaction among lymphocytes, fibroblasts, osteoclasts and osteoblasts drives the bone destruction in RA. IL-17 produced by Th17 cells induces RANKL expression in synovial fibroblasts. Th17 cells induce the proinflammatory cytokines including TNF, IL-6 and IL-1, which further upregulate RANKL expression. Synovial fibroblasts in RA consist of two main types: inflammatory fibroblasts in the sublining layer and RANKL^+^ tissue-destructive fibroblasts in the lining layer. The polarization of tissue-destructive synovial fibroblasts is controlled by the transcriptional factor ETS1. The immunoglobulin immune complexes directly promote osteoclastogenesis. Desialylated immune complexes are particularly effective in this process, regulated by an IL-23–Th17 cell-dependent mechanism. TNF induces the production of Wnt inhibitors like DKK1 and sclerostin, suppressing bone formation

Rheumatoid arthritis (RA) is one of the most representative skeletal disorders triggered by an abnormal immune activation. In RA, autoimmune synovial inflammation causes bone and cartilage destruction in the joints. In the inflamed synovium, IL-17-producing helper T cells, Th17 cells, induce the production of the inflammatory cytokines such as TNF, IL-6 and IL-1, thereby promoting local inflammation. In addition to these proinflammatory cytokines, IL-17 produced by Th17 cells stimulates synovial fibroblasts to induce RANKL expression, leading to abnormal osteoclast activation [32, 33]. The synovial fibroblasts in RA can be divided into two major types: inflammatory fibroblasts located in the sublining layer that secrete the inflammatory cytokines and chemokines, and tissue-destructive fibroblasts in the lining layer that express RANKL and matrix metalloproteinases (MMPs) [34]. NOTCH ligands by endothelial cells, TNF and leukemia inhibitory factor (LIF) contribute to the polarization of inflammatory synovial fibroblasts [35–37]. On the other hand, the transcription factor ETS1 drives the polarization toward tissue-destructive fibroblasts by controlling gene expression of RANKL and MMPs [38]. B lymphocytes also contribute to bone loss in arthritis. Immune complexes act directly on Fcγ receptors on osteoclasts to promote the osteoclastogenesis [39, 40]. Desialylated immune complexes are highly effective in promoting osteoclast differentiation [39], and sialylation of IgG-Fc is controlled by an IL-23–Th17 cell-dependent process [41]. Furthermore, RANKL produced by plasma cell is involved in periarticular bone loss, an osteoporotic lesion observed in the bone adjacent to joints in RA [42]. Recent studies using mouse models of RA has revealed that a distinct population of CX3CR1^hi^Ly6C^int^F4/80^+^MHC-II^+^ macrophages function as osteoclast precursors in arthritis joints [43]. Bone marrow-derived circulating monocytes migrate to the synovium and then differentiate into the unique osteoclast progenitors in arthritic conditions. Osteoblastic bone formation is also affected by the immune factors in arthritis joints. In the synovium, TNF induces the production of the Wnt inhibitor Dickkopf-related protein 1, leading to suppression of bone formation [44]. Sclerostin, another inhibitor of the Wnt signaling pathway, is also elevated in the synovium [45]. Sclerostin-neutralizing antibody inhibited periarticular and systemic bone loss in the mouse models of RA [46], whereas genetic *Sost* (encoding sclerostin) deletion aggravated joint inflammation and destruction in TNF transgenic mice because sclerostin blocks signaling pathways downstream of TNF [45]. Collectively, the complex immune-bone crosstalk among lymphocytes, synovial fibroblasts, osteoclasts and osteoblasts in arthritic joints underlies the pathogenesis of bone destruction in RA (Fig. 2).

### Immune-mediated bone regulation in other diseases

IL-17-type immune responses also contributes to the pathogenesis of other inflammatory bone diseases. In periodontitis, IL-17, IL-6 and neutrophils-derived oncostatin M induced RANKL in osteoblasts and periodontal ligament cells, leading to periodontal bone destruction [23, 47]. In a mouse model of ankylosing spondylitis (AS), unique IL-23R^+^ T cells expressing IL-17 and IL-22 induced ossification of the tendon–ligament attachment [48]. Studies of human AS patients have shown the pathological significance of IL-17 production by invariant NKT cells, γδ T cells and innate lymphoid cells [49]. In mouse models of chronic IL-17A-mediated skin inflammation, which mimics the psoriatic arthritis, IL-17 from γδ T cells, ILCs and keratinocytes negatively regulated osteogenesis by suppressing the Wnt signaling [50]. In a mouse model of osteoarthritis (OA) induced by anterior cruciate ligament transection, IL-17 produced by Th17 and γδ T cells triggered the senescence in the articular cartilage, and senescent fibroblasts in the joints further promoted Th17 skewing, indicating the bidirectional connection between IL-17-type immunity and cell senescence in OA [51]. On the other hand, studies on a mouse model using osteosarcoma cell line AX have shown that TNF by tumor-associated macrophages and IL-17 produced by CD4^+^ lymphocytes maintain osteosarcoma cells in an undifferentiated state by inhibiting their osteoblastic differentiation [52, 53]. The findings suggested that inhibitory effects of IL-17 and TNF on osteoblastogenesis contribute to osteosarcoma progression. Unlike IL-17, IFN-γ, which is a key cytokine of Th1 cells and cytotoxic CD8 T cells, acts as a potent inhibitor of osteoclast differentiation [2, 32]. Mendelian susceptibility to mycobacterial disease (MSMD), a primary immunodeficiency caused by IFN-γR1, IFN-γR2 or signal transducer and activator of transcription (STAT) 1 deficiency, leads to vulnerability to intramacrophagic pathogens and osteomyelitis. In MSMD patients, defective IFN-γ-STAT1 signaling results in abnormal activation of osteoclasts, possibly causing multifocal osteomyelitis [54]. Therefore, it is considered that the physiological significance of IFN-γ-mediated suppression of osteoclasts might be to limit excessive bone resorption during infections.

### Immuno-bone crosstalk in bone healing

The immune cells also play critical roles in the bone regeneration process after the injury. Immediately after bone fracture, blood vessels at the fracture site get ruptured causing bleeding, leading to the accumulation of lymphocytes and macrophages that triggers inflammation. IL-17-producing γδ T cells accumulating at the fracture site stimulate directly PDGFRα^+^Sca1^+^ mesenchymal progenitor cells, thereby inducing cell proliferation and osteoblast differentiation [55]. On the other hand, effector memory CD8 T cells negatively regulated bone regeneration by inhibiting the osteogenesis of mesenchymal progenitor cells via IFN-γ and TNF [56]. Bone fractures in the elderly tend to heal more slowly than those in younger patients. Studies on mouse models of bone fracture showed that the age-related decline in bone repair was attributed to a decrease in macrophage-produced low-density lipoprotein receptor-related protein 1 (Lrp1) that rejuvenate fracture repair [57]. Immune cells exhibit different outputs to bone remodeling by acting on osteoclasts, osteoblasts, fibroblasts and mesenchymal stem cells, depending on the physiological and pathological context [58].

## Regulation of the immune system by bone: bone marrow hematopoiesis

**Fig. 3:**
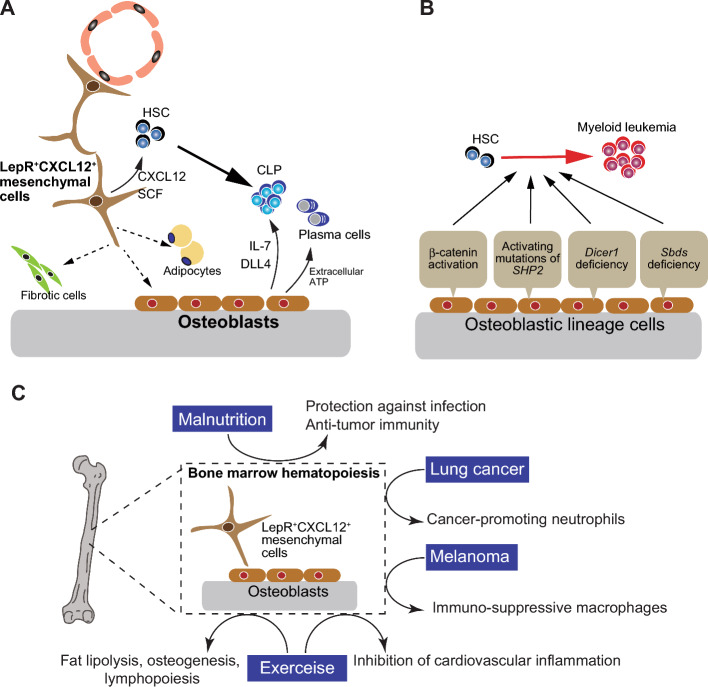
Immune regulation by bone cells in the bone marrow microenvironment **A** LepR^+^CXCL12^+^ mesenchymal stem cells support HSCs by producing key factors SCF and CXCL12. LepR^+^CXCL12^+^ mesenchymal stem cells have the capacity to differentiate into osteoblasts and adipocytes in adult bone marrow, and fibrotic cells in the pathological settings. Osteoblasts maintains common lymphoid progenitors by producing IL-7 and DLL4, and plasma cells via extracellular ATP in the bone marrow. **B** Alterations in osteoblasts result in the development of myeloid leukemia. Activation of β-catenin and SHP2 in osteoblasts induces myeloid leukemia. Deletion of Dicer1 in osteoblasts leads to myelodysplasia and leukemia, through decreased expression of the *Sbds* gene, which is frequently mutated in Shwachman–Diamond syndrome. **C** Various body stresses, including tumors, exercise and nutrition deprivation, stimulate osteoblasts and stromal cells in the bone marrow microenvironment, resulting in altered immune cell differentiation. The effects extend to immune responses in extraskeletal tissues, influencing anti-tumor immune responses, infection protection and inflammatory responses

In the bone marrow, mesenchymal lineage cells build up the specialized microenvironment for the maintenance of HSC and the progeny. LepR^+^CXCL12^+^ mesenchymal stem cells serve as the HSC niche with the high level of production of SCF and CXCL12, both of which are required for HSC maintenance and retention [59]. Notably, LepR^+^CXCL12^+^ mesenchymal stem cells are a main source of osteoblasts and adipocytes in adult bone marrow [60], and can undergo fibrotic differentiation under pathological conditions (Fig. 3A). On the other hand, osteoblasts maintain common lymphoid progenitors in the bone marrow by producing IL-7 and DLL4 [61, 62]. Recently, it has been shown that osteoblast-derived extracellular ATP is required for plasm cell maintenance in the bone marrow [63] (Fig. 3A). Changes in the osteoblastic status critically interfere with normal hematopoiesis. In sepsis, increased G-CSF causes a drastic reduction of osteoblasts in the bone marrow, resulting in a decrease in common lymphocyte progenitor cells and lymphopenia [61]. Activation of β-catenin in osteoblasts induced acute myeloid leukemia [64], and activating mutations of the protein tyrosine phosphatase SHP2 resulted in the development of leukemia [65]. Deletion of Dicer1 in osteoblasts led to decreased expression of the *Sbds* gene, which functions in ribosome synthesis, leading to myelodysplasia and leukemia [66]. The *SBDS* gene is frequently mutated in Shwachman–Diamond syndrome, and *Sbds*-deficient mesenchymal cells induced genotoxic stress in HSCs via S100A8/9 production [67]. Osteoblastic lineage cells are essential for the proper maintenance of lymphoid progenitors in the bone marrow, while their alteration can lead to hematological malignancies (Fig. 3B).

Since osteoclasts form the bone marrow cavity, their involvement in the regulation of HSCs has also been examined as a topic of significance. Osteoclasts secrete MMP9 and cathepsin K to degrade CXCL12, thereby abrogating the HSC retention to the niche cells [68], suggesting that osteoclastic bone resorption promotes HSC mobilization from the bone marrow. However, G-CSF-induced HSC mobilization was not suppressed but rather enhanced in mice treated with alendronate or neutralizing RANKL antibody, and osteoclast-deficient mice including *op/op*, c-Fos-deficient and RANKL-deficient mice [69]. Consistent with the findings, it was reported that treatment with pamidronate or OPG-Fc did not reduce HSC mobilization to the periphery [70, 71]. On the other hand, osteoclast inhibition resulted in a decrease in the frequency and number of HSCs and B cells through the modulation of the bone marrow mesenchymal cells [72, 73]. It was also reported that calcium-sensing receptor on HSCs is required for their homing to the endosteal niche, suggesting that high Ca^2+^ level resulting from osteoclastic bone resorption is involved in HSC maintenance [74]. Therefore, osteoclast may play a role in HSC regulation in the bone marrow under in some cases, but at least they are less involved in G-CSF-induced HSC mobilization.

### Impacts of osteoporosis on the immune system

Given that osteoclasts and osteoblasts are somehow involved in HSC regulation in the bone marrow, it would be possible that the immune system is influenced by the osteoporotic conditions. The severe osteoporosis caused by OPG-deficiency resulted in decreased G-CSF-induced mobilization of HSCs [69]. Together with the findings that HSC mobilization was increased by pharmacological or genetic inhibition of osteoclast activity as described above, osteoclast activity might negatively regulate HSC mobilization. Human studies on postmenopausal osteoporosis have reported that the number of several memory B cell subsets were significantly lower in women with osteoporosis and positively correlated to bone mineral density [75]. Bioinformatics analysis using expression profiles of bone samples derived from postmenopausal osteoporosis patients suggested that the frequencies of T and NK cells increases during the progression of postmenopausal osteoporosis [76]. In addition, the expression level of the osteoclastogenic cytokines such as IL-17, TNF and IL-6 were upregulated by estrogen deficiency [77]. These findings suggested that the immune cell development or activation status can be affected by postmenopausal osteoporosis. Furthermore, it was reported that ovariectomy caused gut permeability to induce Th17 cell expansion in the gut, which contributes to bone loss [78]. However, the role of lymphocytes in estrogen deficiency-induced bone loss remains controversial, since neither TCRα-deficient nor Rag2-deficent mice display any obvious bone phenotype. Since factors causing osteoporosis such as sex hormone changes, aging and autoimmunity can directly affect the immune system, caution is needed to determine the causal relationship between bone loss and immune cell changes in osteoporosis.

### Aging-related immune–bone crosstalks in the bone marrow

Bone structure and mass change with age, characterized by decreased bone remodeling, decreased bone mass, increased bone porosity, cortical thinning, expansion of bone marrow fat and delayed bone regeneration [79, 80]. In addition, aging also affects hematopoiesis in the bone marrow, which shows a bias toward myeloid differentiation with aging [81]. The myeloid skewing during aging was reported to result from skeletal stem cell aging [79], and IL-1 production by osteoprogenitors and arteriolar endothelial cells [82]. Skeletal stem cell aging was also implicated in aging-impaired skeletal regeneration [79]. Conversely, grancalcin production by aged neutrophils and macrophages promoted skeletal aging including low bone formation and marrow fat accumulation [83]. Recently, light-sheet imaging of the skeletal tissues has revealed lymphatic vessels in bones, which contributed to bone and hematopoietic regeneration after stress [84]. Aging led to impairment in stress-induced lymphatic expansion in the bone marrow, reducing the regeneration abilities in the bone. Aging significantly alters the mode of crosstalk between bone and the immune system in the bone marrow microenvironment.

### Changes in bone marrow microenvironment by exercise, malnutrition and tumors

Physical activity affects the bone marrow environment, modulating the immune cell differentiation. Exercise-induced reduction of leptin production in adipose tissue promoted CXCL12 expression in LepR^+^ mesenchymal stem cells in the bone marrow, keeping HSCs in a quiescent phase [85]. As a result, inflammatory leukocyte decreased, contributing to attenuation of cardiovascular inflammation. Exercise induced the secretion of reticulocalbin-2 from bone marrow macrophages [86]. Reticulocalbin-2 enhanced bone marrow fat lipolysis, leading to osteogenesis and lymphopoiesis.

Nutritional status also has a profound effect on the immune system by altering the bone marrow environment. Nutrient deprivation increased CXCL13 production in the bone marrow, which induced migration of naive B cells from intestinal Peyer’s patches (PP) into the bone marrow [87]. Naïve B cells swiftly migrated back to PP in response to refeeding, and thus repeated fasting impacted B cell dynamics, resulting in attenuated antigen-specific mucosal immune responses. In addition, dietary restriction increased memory T cell accumulation in the bone marrow via adipocytes, CXCR4-CXCL12 and S1P-S1P1R [88]. Malnutrition-induced homing of memory T cells to bone marrow was associated with enhanced immunity against infection and tumors [88].

Tumor cells can escape the immune attack by establishing the microenvironment that suppresses anti-tumor immune responses by incorporating immune-suppressive cells and factors. Interestingly, tumors not only act locally on immune cells around tumor tissues, but also stimulates the osteoblastic cells in the bone marrow, thereby eliciting the development of immune-suppressive cells. Lung cancer-derived soluble receptor for advanced glycation end products (sRAGE) remotely activated osteoblasts in bones. In turn, osteoblasts supply lung tumor with pro-tumorigenic SiglecF^high^ neutrophils, resulting in tumor progression [89]. Skin cancer remotely acts on osteoblasts and osteocytes to promote DKK1 production. DKK1 induced the expansion of myeloid-derived suppressor cells, which suppresses the anti-tumor immune response [90]. Through the modulation of bone marrow osteoblasts and stromal cells organized into the hematopoietic niche, various pathophysiological stresses including aging, physical exercise, malnutritional and tumors substantially impact the immune responses (Fig. 3C).

## Concluding remarks

The review outlines the impact of the immune system on bone in the context of tissue repair and destruction, and the regulation of the immune cell differentiation by bone cells in the bone marrow, shedding light on the bidirectional reciprocal relationship between the two systems. The findings gained from osteoimmunology research have greatly contributed to the clinical and drug discovery fields, particularly in the development of therapeutic agents for RA. Elucidation of the effects of immune cells/factors on bone has led to the understanding of the mechanism of action of drugs such as immune-modulating biologics on bone destruction in arthritis. On the other hand, much knowledge has been accumulated about immune regulation by bone cells in the bone marrow, and its significance in various physiological and pathological settings, such as aging and cancer, is becoming clear. The next step for osteoimmunology would be how to feed these findings back into clinical practice, for example, in the prevention and treatment for cancer and aging-related disorders as well as various diseases of bone and/or the immune system. Recent advances in single-cell omics technology have made it possible to analyze the heterogeneous immune cell populations at high resolution, even in human peripheral blood and tissue samples. The technique of human immune phenotyping has been applied in various clinical studies, and human immunology is making great progress. However, it is still difficult to decipher the multicellular dynamics of human bone and bone marrow at the single-cell level. In addition to LepR^+^CXCL12^+^ bone marrow mesenchymal stem cells, novel types of mesenchymal stem cells such as periosteal stem cells and growth plate resting zone stem cells, vertebral skeletal stem cells have recently been discovered, further spotlighting the extremely heterogeneous nature of mesenchymal cells in bone. In deciphering the immune–bone crosstalk in the bone marrow, we must keep in mind that the bone marrow microenvironment is composed of an extremely diverse population of cells belonging not only to bone metabolism and the immune system, but also to nervous and vascular systems. Further development of multi-omics and imaging techniques would offer a comprehensive view of diverse cellular dynamics in bone, enhancing our understanding of the complex regulatory mechanisms that govern the interplay between the skeletal and immune systems.
