# Correction: Crosstalk between bone and the immune system

**DOI:** 10.1007/s00774-024-01547-x

**Published:** 2024-09-06

**Authors:** Kazuo Okamoto

**Affiliations:** 1 Department of Osteoimmunology, Graduate School of Medicine and Faculty of Medicine, The University of Tokyo, Tokyo, Japan; 2 Division of Immune Environment Dynamics, Cancer Research Institute, Kanazawa University, Kakuma-Machi, Kanazawa, 920-1192 Japan

**Correction: Journal of Bone and Mineral Metabolism** 10.1007/s00774-024-01539-x

In this article the top of Fig. 2 is cut off; the Fig. 2 should have appeared as shown below.

**Fig. 2:**
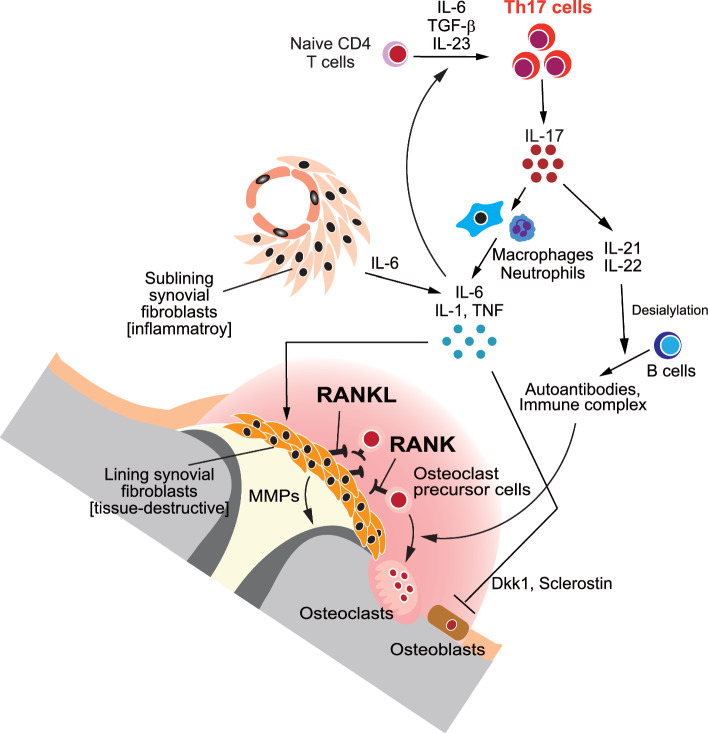
Mechanism of bone destruction in RA. The intricate immune–bone interaction among lymphocytes, fibroblasts, osteoclasts and osteoblasts drives the bone destruction in RA. IL-17 produced by Th17 cells induces RANKL expression in synovial fibroblasts. Th17 cells induce the proinflammatory cytokines including TNF, IL-6 and IL-1, which further upregulate RANKL expression. Synovial fibroblasts in RA consist of two main types: inflammatory fibroblasts in the sublining layer and RANKL^+^ tissue-destructive fibroblasts in the lining layer. The polarization of tissue-destructive synovial fibroblasts is controlled by the transcriptional factor ETS1. The immunoglobulin immune complexes directly promote osteoclastogenesis. Desialylated immune complexes are particularly effective in this process, regulated by an IL-23–Th17 cell-dependent mechanism. TNF induces the production of Wnt inhibitors like DKK1 and sclerostin, suppressing bone formation

The original article has been corrected.

